# Experimentally derived model shows that adaptation acts as a powerful spatiotemporal filter of visual responses in the rat collicular neurons

**DOI:** 10.1038/s41598-018-27331-2

**Published:** 2018-06-12

**Authors:** Juntaute Bytautiene, Gytis Baranauskas

**Affiliations:** 1 Faculty of Medicine, Lithuanian University of Health Sciences, Kaunas, 50161 Lithuania; 2 Neurophysiology laboratory, Neuroscience Institute, Lithuanian University of Health Sciences, Kaunas, 50161 Lithuania

## Abstract

Adaptation of visual responses enhances visual information processing mainly by preserving the full dynamic range of neuronal responses during changing light conditions and is found throughout the whole visual system. Although adaptation in the primate superior colliculus neurons has received much attention little is known about quantitative properties of such adaptation in rodents, an increasingly important model in vision research. By employing single unit recordings, we demonstrate that in the rat collicular neurons visual responses are shaped by at least two forms of adaptation. When visual stimuli were repeatedly presented in the same location, visual responses were reduced in the majority of single units. However, when the adaptor stimulus was outside a small diameter receptive field (RF), responses to stimulus onset but not offset were enhanced in the majority of units. Responses to stimulus offset were reduced less and recovered faster than responses to stimulus onset and the effect was limited to a fraction of RF area. Simulations showed that such adaptation acted as a powerful spatiotemporal filter and could explain several tuning properties of collicular neurons. These results demonstrate that in rodents the adaption of visual responses has a complex spatiotemporal structure and can profoundly shape visual information processing.

## Introduction

In early days of vision science the term ‘adaptation’ was usually associated with a decreased amplitude of responses to repeated visual stimulus presentations^1–3^. However, recent evidence shows that this simple form of non-associative learning is not limited to suppressive effects and its main purpose is, probably, to efficiently convey information about visual objects^4^. In the retina adaptation to light helps to maintain the full range of neuronal responses under changing levels of illumination^5^. To prevent response saturation when stimulus brightness increases, adaptation in the retina reduces the response amplitude. However, if light levels drop, response amplitude will recover and neurons will be able to respond to much smaller changes in illumination. Although at later stages of the visual pathway adaptation may serve similar purpose^6,7^, there is evidence for other functions as well, such as salience modulation, increased efficiency in sensory representation and spatial integration^4^.

A simple form of adaptation manifested as a decrease in the response magnitude during repeated visual stimulus presentations has been reported in the first studies of the superior colliculus (SC) neurons^1,2,8^. Research in monkey SC revealed that even a small adaptor stimulus, covering only a fraction of the receptive field (RF) of the neuron, affected responses to any subsequent stimulus placed within RF of that neuron^9^. Both the onset and the recovery from adaptation in monkey SC is very fast: light flashes lasting only 17 ms could induce a 50% reduction in the number of evoked action potentials and the responses recovered to nearly original magnitude in less than half a second^9–12^. Rapid recovery from adaptation ensures that even frequent presentation of visual stimuli, one each second, will result in little or no adaptation. However, the absence of quantitative data on the onset speed of adaptation in monkeys does not permit to accurately predict adaptation effects on complex stimuli. Surprisingly, there is virtually no quantitative information on adaptation in rodent SC in spite of the fact that adaptation may affect visual responses to visual stimuli presented too frequently during experiments. Even though a number of papers mention the presence of adaptation of visual responses in rodent collicular neurons, none of them report any numbers on temporal or spatial features of adaptation^1,8,13,14^. Rodents, especially mice are increasingly becoming animals of choice for vision research studies because of availability of molecular tools^15–17^. Although many molecular biology techniques were less successful in rats, these large rodents exhibit rich behavior, are more accessible for multielectrode recordings and an increasing number of modern techniques, including optogenetic methods are employed in the rat research^17–22^. Therefore we performed a quantitative study of adaptation of visual responses in the rat superior colliculus superficial layer (SCS) neurons. Our data show that, in contrast to visual responses in monkey^9^, both local and non-local forms of adaptation affect visual responses of rat SCS neurons resulting in a powerful spatiotemporal filter. Most adaptation in SCS neurons is likely to be inherited from the retinal light adaptation while the off-RF effects are explained by the normalization model of adaptation^4^. Our quantitative data on temporal and spatial properties of adaptation allowed us to build a model, which could predict the adaptation effects on complex stimuli such as moving gratings.

## Results

### The extent of adaption of visual responses in the rat collicular neurons

We used adult (>6 weeks old, n = 23) Wistar rats for this study. All recorded single units (n = 64) were less than 300 μm deep from the SC surface, corresponding to the superficial layers of SC. Most rat SC neurons respond well to small round spots^8,23–25^ but poorly to gratings of spatial frequencies above ~0.5 cycle per degree^26^. Therefore, to achieve sufficient spatial resolution, round bright spots were used as visual stimuli. In addition, the use of spots enabled us to easily separate ON and OFF responses, occurring correspondingly after the appearance and the disappearance of a bright spot^1,25^. Experiments described below show that there are several major differences in adaptation of ON and OFF responses.

**Figure 1:**
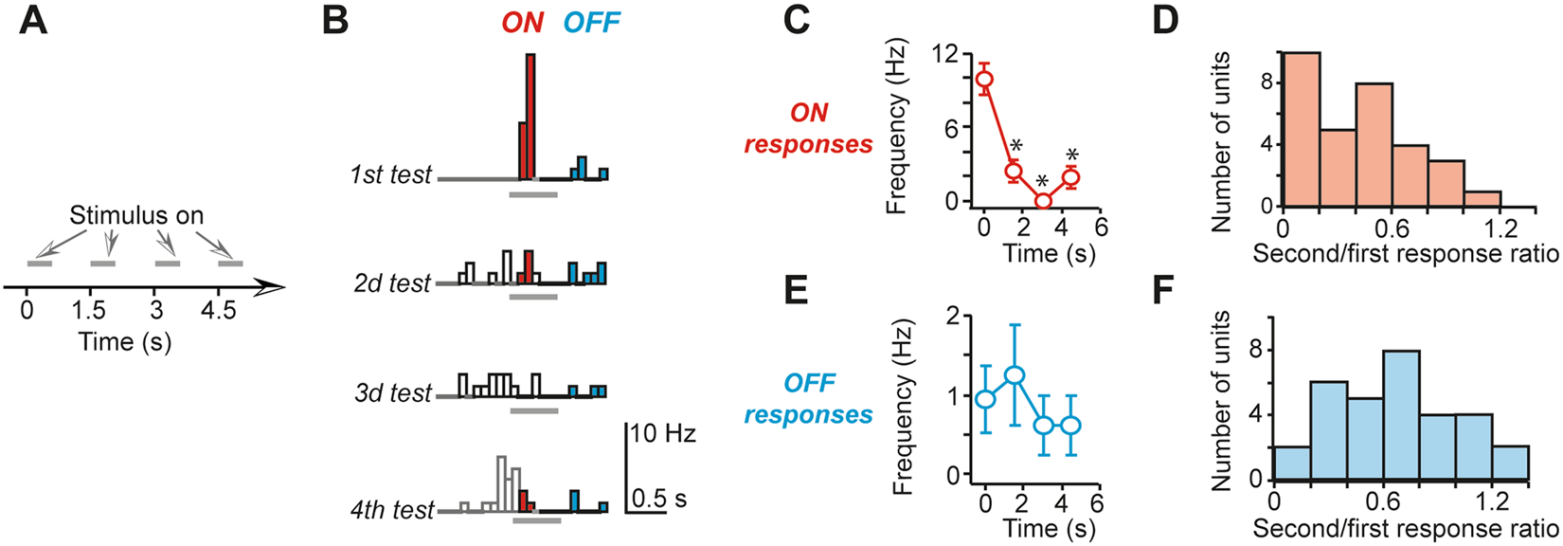
In rat collicular neurons responses to visual stimuli gradually diminished during repeated presentations. (**A**) The schematics of stimulation protocol. (**B**) Peristimulus time histograms (PSTHs) of response to multiple presentations. Red and blue bars correspond to bins used to calculate ON and OFF responses correspondingly that occur during stimulus onset (‘ON’) and offset (‘OFF’). 8 tests were used to build the histograms. (**C**) The average action potential frequency during ON responses of a single unit shown in (**B**). Each open circle corresponds to an average AP frequency during ON response to a stimulus presented at a time point shown on the horizontal axis. (**D**) The distribution among recorded units of 2d/1st response AP frequency ratio. (**E**,**F**) The same as C and D correspondingly but for OFF responses.

Having identified the receptive field (RF) of a unit by flashing 2.5°–3.0° round spots randomly on a 11 × 18 grid at 10° spacing, the adaptation of responses to repeated visual stimulus presentations was first observed by flashing 4–6 times 2.5° spots inside RF for 0.6 s at 1.5 s inter-stimulus interval (ISI), corresponding to a 0.9 s gap between stimuli (Fig. 1A). In about 1/3d of units a short burst of action potentials (APs) induced by the first stimulus presentation was followed by single or no AP responses to the subsequent stimulus presentations in spite of an increase in the background activity due to summation of slow OFF responses (Fig. 1B). Although such a dramatic reduction in AP frequency was not observed in all units, on average the AP frequency of the second ON response was reduced by 60 ± 6% (n = 31, p < 0.00001, Wilcoxson signed ranks test, Fig. 1D). A statistically significant reduction in the ON response AP frequency, determined by a non-parametric Kruskal-Wallis test at a significance level p < 0.05, could be detected in 25 out of 31 units (81% of units, up to 6 presentations were used for statistical analysis). Usually, the AP frequency during OFF responses was reduced much less (p = 0.0013, Wallace approximation in the Kruskal-Wallis test, n = 31 for ON responses and n = 30 for OFF responses, Fig. 1E). On average the AP frequency of the second OFF response was reduced by 32 ± 6% (n = 30, p = 0.004, Wilcoxson signed ranks test, Fig. 1F). A significant reduction in the OFF response AP frequency was observed in 19 out of 30 units (63% of units). It should be noted that our stimulation protocol may have affected the extent of OFF response sensitivity to adaptation: in contrast to ON responses, OFF responses were always preceded by a test stimulus that itself may have induced adaptation. In other words, our control OFF but not ON responses may be already adapted. Nevertheless, data provided below clearly show that there are differences between ON and OFF response adaptation that cannot be explained by this stimulation difference. These results confirm previous reports that SCS neurons in rodents are susceptible to adaptation although we are unaware of any quantitative study^1,14^. Since rodents are increasingly used for vision research, we performed experiments to obtain adequate quantitative description of adaptation in rat SC neurons.

### Temporal characteristics of adaptation in the rat SC

**Figure 2:**
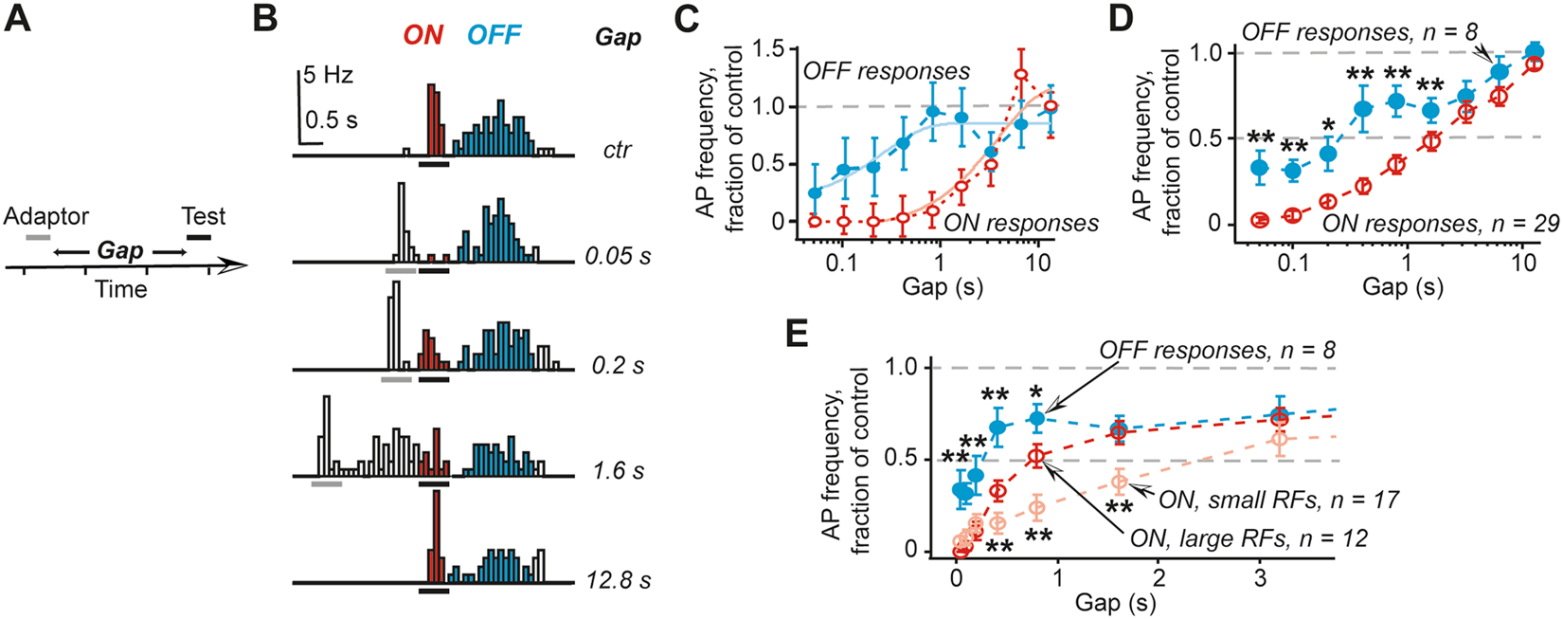
Recovery analysis reveals differences between ON and OFF responses and the units with large and small RFs. (**A**) Schematics of the stimulation protocol used in these tests, the protocol was repeated each 30 s. (**B**) Non-subtracted PSTHs of a representative unit are shown for responses to stimuli presented without the adaptor stimulus (control) and following 0.05 s, 0.2 s, 1.6 s and 12.8 s gap after the adaptor stimulus. (**C**) The recovery of ON (blue circles) and OFF (red circles) responses of the unit shown in (**B**,**D**) Summary of all tested units. (**E**) Summary of ON response recovery for small RF ((>20°) and large RF (>20°) units, OFF response recovery was the same for all units and it is added for comparison. Note that in E the time scale is linear but not logarithmic as in (**C**,**D**). In (**D**,**E**) asterisks indicate statistically significant differences between ON and OFF responses in *D* and between OFF and large RF or large and small RF units in *E* for the same gap duration: **indicates p < 0.01 while *corresponds to p < 0.05.

Any experiment involving repeated tests may result in adaptation of responses. Therefore, first it was necessary to establish a safe interval between tests that would not cause significant adaptation effects. Early papers on adaptation of visual responses in rat SC neurons suggested that stimuli up to few seconds long may be required to induce clear effects while a gap of >10 s was necessary to recover the original response amplitude^13,14^. Therefore, first we tested whether 10 s were sufficient to recover adapted responses. To this end, every 90 s a conditioning stimulus of 0.6 s was followed by a test stimulus of the same duration at 1.5–30 s ISI (Supplementary Fig. 1). These experiments confirmed, that 11 s gap was sufficient to recovery most responses: on average the ON response AP frequency was 95 ± 5% of the control (range 76% to 125%, n = 10, p > 0.1, Wilcoxson signed ranks test) while the OFF response AP frequency was 97 ± 6% of the control (range 72% to 122%, n = 9, p > 0.1, Wilcoxson signed ranks test) after 11 s gap. There was no single unit in which the ON or OFF response AP frequency would differ significantly from control after an 11 s gap. Therefore, to determine the time-course of recovery from adaptation in detail, the stimulation protocol was repeated each 30 s while the last test stimulus was followed by at least 15 s gap (Fig. 2A,B).

It should be noted that so far we did not take into account the overlap of responses to adaptor and test stimuli because it was a minor issue for relatively long gaps of >0.9 s used in the above described experiments. However, to generate test data shown in Fig. 2B gaps as short as 50 ms were employed and it was necessary to subtract the response to the adaptor stimulus from the response to the test stimulus (Supplementary Fig. 2). The time-course of recovery for both ON and OFF responses shown in Fig. 2C was obtained following this subtraction procedure. It is an example of a unit, in which no or almost no ON responses were observed for brief gaps of <0.2 s. The recovery of OFF responses was much faster that was a general pattern followed in the majority of units (Fig. 2C,D). In addition, there was a significant difference between the units with small and large RFs. 2/3ds of all units had small RFs (<20°). In these units the recovery from adaptation was significantly slower than in the units, the RF diameter of which exceeded 20° (Fig. 2E). No such difference was found for OFF responses.

**Figure 3:**
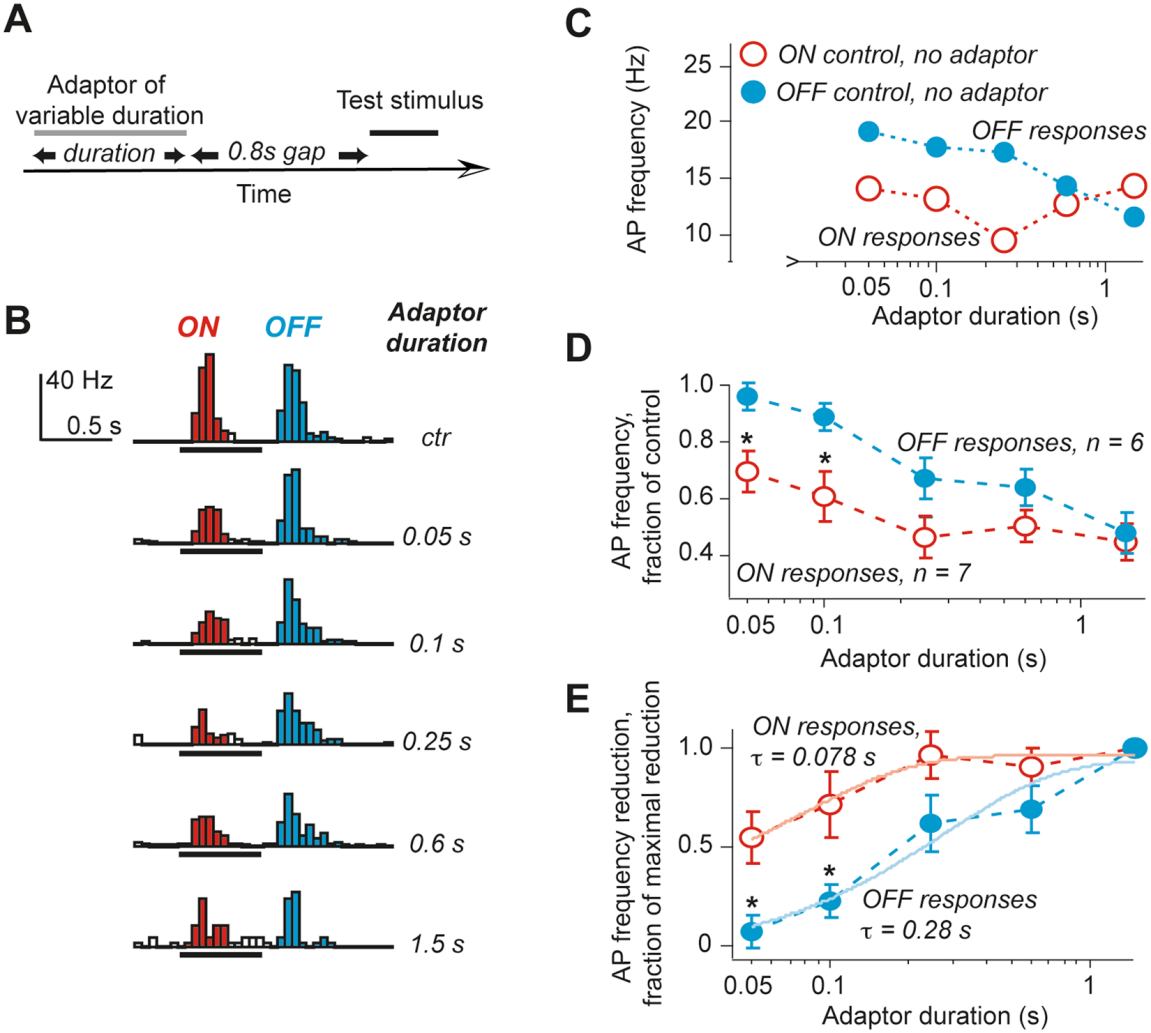
Even a very brief presentation of an adaptor was sufficient to induce long lasting adaptation. (**A**) A schematic representation of stimulation paradigm. (**B**) An example PSTH of responses obtained with adaptors of different duration. Note a clear decrease in the ON response bar amplitude, depicted in red, following a 0.05 s adaptor stimulus. OFF responses (blue PSTH bars) were unchanged following a 0.05 s adaptor. (**C**) The average normalized response amplitudes are plotted as a function of the adaptor stimulus duration. In the left upper corner ON and OFF response amplitude values without adaption are shown. (**D**) The summary of all tested units. (**E**) The same data as in *D* re-plotted as a function of the maximal decrease in the AP rate during responses. Lines represent fits to a mono-exponential function. In (**D**,**E**) asterisks indicate statistically significant differences between ON and OFF responses (p < 0.05).

Having established the time-course of recovery from adaptation, next we determined the time-course of induction of adaptation. To this end, the duration of an adaptor stimulus was varied between 0.05 s and 1.5 s and 3 different gap durations between the adaptor and the test stimulus were examined, 0.1 s, 0.8 s and 6.4 s (Fig. 3A). In 6.4 s gap tests, there was only a minor reduction in the response amplitude confirming our previous results that most recovery from adaptation was nearly complete in <10 s. Meanwhile, the results with 0.1 s and 0.8 s gaps were similar and we show only data for 0.8 s gaps. These experiments revealed that in the majority of units even a 0.05 long adaptor stimulus was sufficient to induce adaptation of ON responses to nearly the same extent as a 0.6 s or 1.5 s adaptor stimuli (Fig. 3B–D). For every single unit tested, we normalized the reduction of the response amplitude to the maximal reduction achieved with a 1.5 s adaptor (Fig. 3E). Then, by fitting the obtained results with a mono-exponential function we were able to estimate the time constant of the adaptation onset. It was 0.078 ± 0.004 s for ON responses and 0.28 ± 0.14 s for OFF responses, more than 3 fold difference.

### Spatial specificity of adaptation in the rat SC neurons

It has been shown that adaptation effects may depend on spatial location within RF^6,27,28^. If adaptation is inherited from the brain area where RF size is much smaller, then adaptation may affect only a small fraction of RF close to the adaptor^6^. SCS neurons receive their main inputs from the retina and the cortex^29^. RF size of rat SC cells may be larger than the RF size found in the retina^8,30–33^; thus adaptation inherited from the retina and induced by a small adaptor may be limited to a fraction of RF close to the adaptor location. Although tests with gratings did not reveal any spatial specificity of adaptation in monkey SC neurons^9^, in rodents spatial properties of adaptation may differ from monkey SC neurons. In fact, the recovery of ON responses described here is much slower than the recovery from adaptation in monkey SC^11,12^. Thus next we investigated spatial properties of slowly recovering adaptation that was present in rat but not monkey SC neurons.

**Figure 4:**
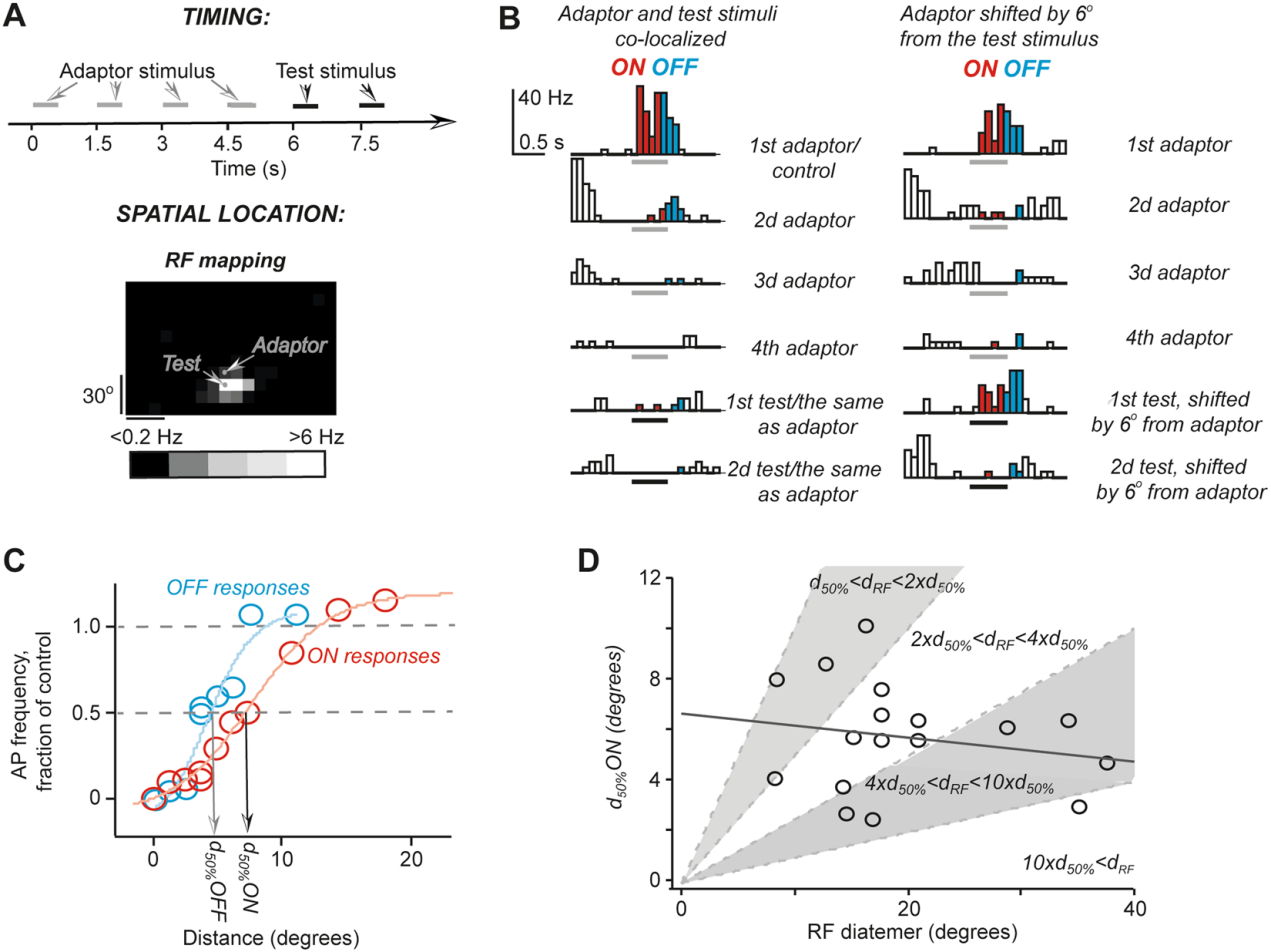
Slow adaptation can be eliminated by shifting the adaptor stimulus within RF. (**A**) A schematic representation of stimulation paradigm. (**B**) An example of responses obtained for co-localized adaptor and test stimuli (left column of PSTHs) and for adaptor presented in location, which was shifted to a distance close to *d*_*50%*_. Scale bar: 40 Hz and 0.2 s. (**C**) The summary of ON and OFF responses of the unit shown in (**B**) Normalized response amplitude is plotted against adaptor location distance from the test stimulus location. (**D**) A plot of *d*_*50%*_ against RF diameter to demonstrate the lack of correlation between RF size and *d*_*50%*_. Each open circle in the plot corresponds to one unit. The graph area is divided into regions of different ranges of RF diameter to *d*_*50%*_ ratios. These ranges are indicated by equations. Note that for all units RF diameter was larger than *d*_*50%*_.

To increase the sensitivity of our tests, a train of 4 adaptor stimulus presentations was followed by two test stimulus presentations, these stimuli were presented each 1.5 s corresponding to 0.9 s gap between two stimuli (Fig. 4A). This paradigm is similar to the one used to generate data of Fig. 1 with one key difference: the adaptor stimulus was presented at a location that was shifted away from the test stimulus location. In a control series, the adaptor and the test stimuli were co-localized; and this run was used to determine the decrease in AP frequency caused by adaptation (Fig. 4B, left column PSTHs): a difference between the AP frequency in response to the 1^st^ and to the 5^th^ stimuli was considered as a fraction of the response sensitive to adaptation. When the adaptor stimulus was shifted away from the test stimulus location resulting in reduced adaptation, the 5^th^ stimulus corresponded to the test stimulus that was shifted from the adaptor location (Fig. B, right column PSTHs). We define *d*_*50%*_ as a visual angle between the test stimulus and the adaptor, at which 50% of the response amplitude sensitive to adaptation was restored (Fig. 4C).

These experiments revealed that most slowly recovering adaptation could be eliminated by placing the adaptor stimulus 10 degrees or more away from the test stimulus location (Fig. 4C). The elimination could be achieved inside RF and usually *d*_*50%*_ was only a small fraction of the RF diameter (Fig. 4D): only in 4 out 19 units *d*_*50%*_ was about the same as the RF diameter (data points in the upper grey quadrant of Fig. 4D). Although there was no statistically significant difference between *d*_*50%*_ of OFF and ON responses (5.4 ± 0.5 degrees, n = 20, and 3.9 ± 0.5, n = 10, p = 0.98, Mann-Whitney two-tailed test), *d*_*50%*_ of OFF responses were more narrowly distributed as indicated by the difference in the Gaussian fit half-width: 0.8 ± 0.1 degrees for OFF responses and 3.8 ± 1.2 degrees for ON responses (Supplementary Fig. 3).

**Figure 5:**
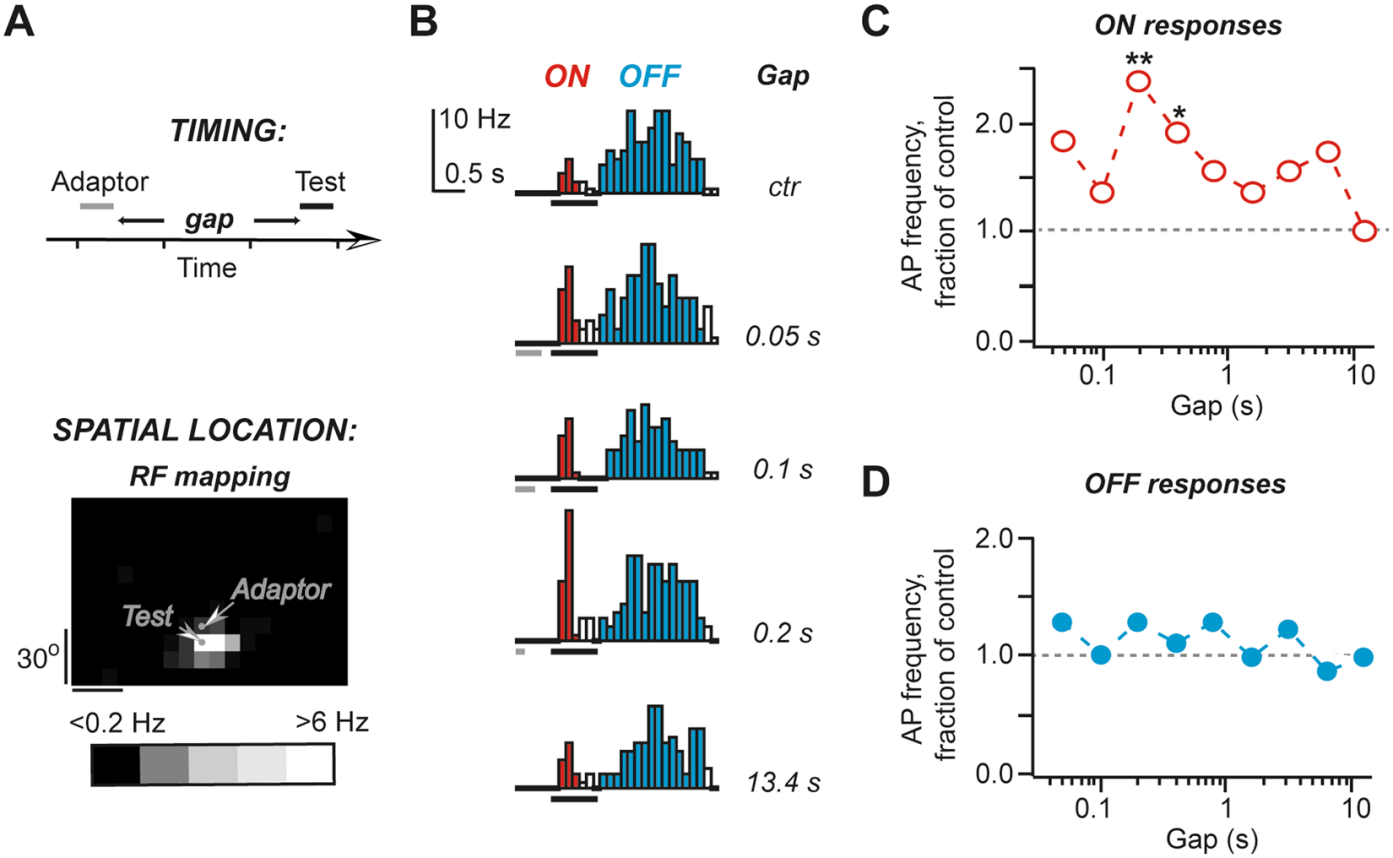
Facilitation can be observed when the adaptor stimulus is placed near small RF. (**A**) A schematic representation of the stimulation paradigm. (**B**) An example of response PSTHs. (**C**) The summary of ON responses obtained for unit shown in (**B**). (**D**) The summary of OFF responses obtained for unit shown in **B**.

**Figure 6:**
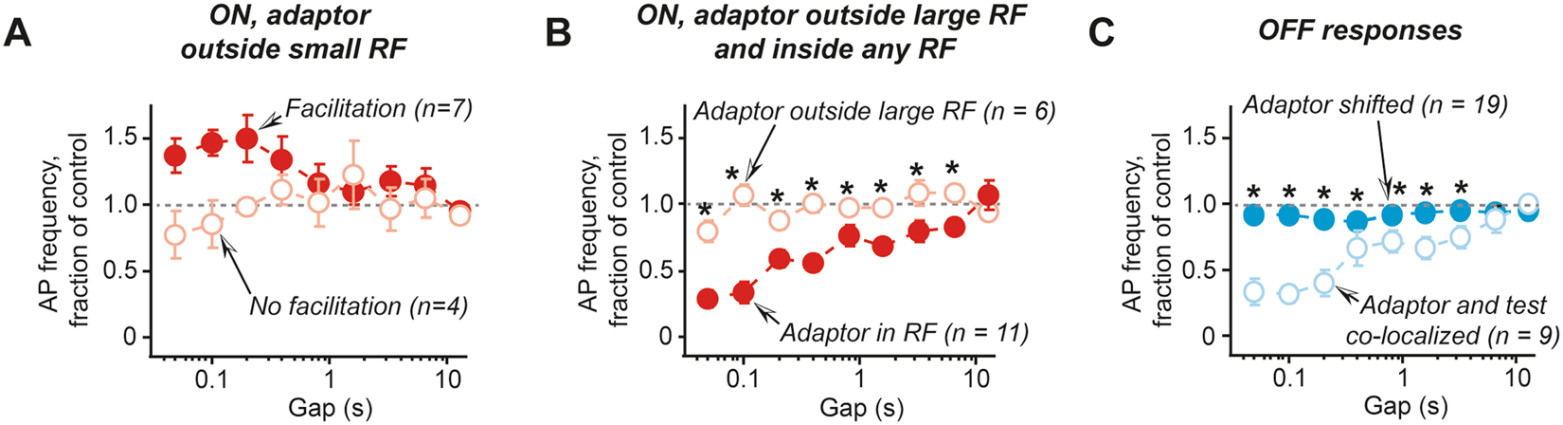
The summary for non-locally induced adaption. (**A**) The average response time-course for all units, which showed significant facilitation for at least one time point when the adaptor was placed close to the small RF (filled circles). For comparison, also units with no facilitation under similar stimulation conditions are shown. (**B**) No facilitation was observed for units with large RF (>20°). The averaged data for all such units with adaptor placed inside RF (filled circles) and outside RF (open circles) are shown. In both cases the adaptor and the test stimuli were >10° apart. (**C**). No adaptation was observed for all OFF responses when the adaptor and the test stimuli were >10° apart (filled circles). For comparison, the average of all OFF responses when the adaptor and the test stimuli were co-localized is also shown (open circles). In (**B**,**C**) asterisks indicate gap durations for which statistically significant differences between two conditions was found (p < 0.05).

Although a 10 degree difference between the adaptor and the test stimulus locations was sufficient to eliminate most slow adaptation, it is plausible that another form of adaptation that recovers rapidly was still present under these conditions. To test this possibility, we tested shorter than 0.9 s gap intervals for adaptor and test stimuli placed >10° from each other (Fig. 5A). The most surprising results were obtained when an adaptor was located just outside small RF as shown in Fig. 5B,C. In this configuration of adaptor and test stimuli, the ON response amplitude was significantly enhanced in a number of units (Fig. 5C). This facilitation of ON responses lasted only few hundred milliseconds. Meanwhile, OFF responses were largely unaffected by the adaptor (Fig. 5D). Such a behaviour of ON and OFF responses was typical. When placed close to a small size RF (<20° in diameter), an adaptor induced statistically significant facilitation for at least one of the tested gap durations in 7 out of 11 units (Fig. 6A, dark red circles). In the remaining 4 units no change in ON response amplitude could be detected (Fig. 6A, light red circles). No such behaviour was observed for units with large RF: for these units, an adaptor outside RF induced no change while an adaptor inside RF resulted in a reduced response amplitude, which recovered very quickly, 50% of the response amplitude was restored in ~0.3 s (Fig. 6B). The response recovery after an adaptor stimulus co-localized with the test stimulus was much slower: to restore 50% of the response amplitude >1 s was required.

In contrast to ON responses, in 19 out of 21 units the amplitude of OFF responses was largely unaffected by an adaptor, displaced by >10° from the test stimulus, with no difference whether the adaptor was inside (n = 9) or outside RF (n = 10). Therefore, to generated Fig. 6C graph, all data were pooled together. Nearly half of these units (n = 9) had significantly reduced OFF response amplitudes when the adaptor and the test stimuli were co-localized, indicating that we did not accidently sampled the units, the OFF responses of which were insensitive to adaptation.

### Evaluation of adaptation functional consequences by employing simulations

**Figure 7:**
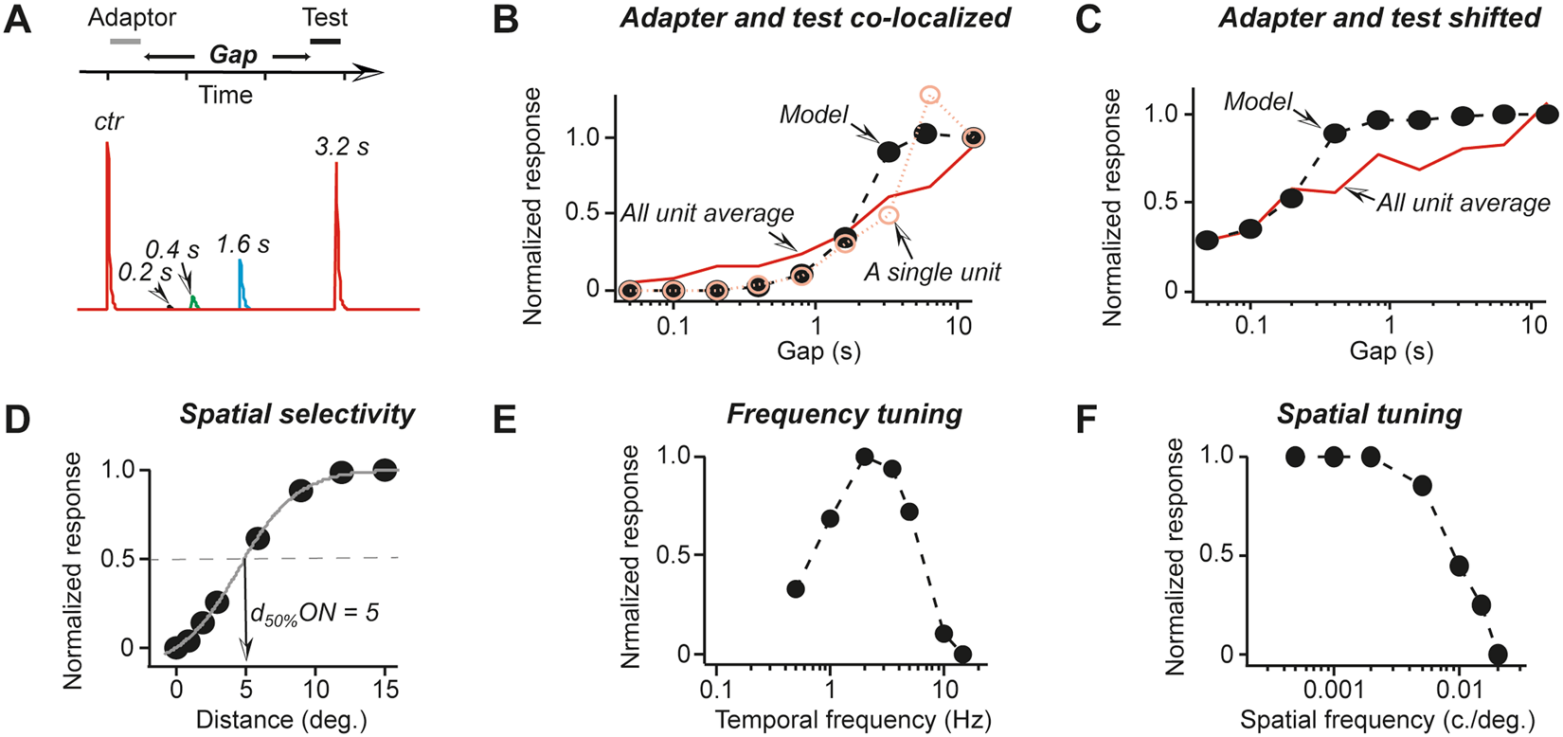
Simulations show profound effects of adaptation on visual responses of SC neurons to gratings. (**A**) An example of responses generated by the adaptation model. The stimulation protocol is shown above, the adaptor and the test stimuli were co-localized. Response traces for the following gap values are overlaid: 0.05 s, 0.1 s, 0.2 s, 0.4 s, 0.8 s, 1.6 s and 3.2 s. Only in the last 4 traces the test stimulus induced a response. (**B**) The summary of simulation results for co-localized adaptor and test stimuli as shown in *A*. Model response amplitude is plotted against gap duration (black filled circles). For comparison, the average data from all units is shown as a red line while data of a single unit, that are displayed in Fig. 4, are shown as light red open circles. (**C**) Simulation data for adaptor and test stimulus placed 14 degrees apart (black filled circles). Experimental data, the average of all units for adaptor and test stimuli set apart are shown as a red line. (**D**) Simulated response data for different distances between the adaptor and test stimuli inside RF are shown as black circles. A grey line is a Sigmoid function fit. (**E**) A plot of simulated response amplitude against the temporal frequency of gratings. The spatial frequency of all gratings was fixed at 0.01 c./deg. (**F**) A plot of simulated response amplitude against the spatial frequency of gratings. The temporal frequency of all gratings was 2 Hz.

To evaluate potential functional consequences of adaptation, a simple model was constructed. To account for both local and non-local adaptation within RF, the model employed the canonical normalization equation^34^. Adaptation effects were introduced by back-feeding a low-pass filtered local and non-local light signals into the gain control stage, similarly to the majority of retinal light adaptation models^35–38^ (details in the Supplementary Materials). For simplicity, the model reproduced only ON response adaptation effects, no OFF responses were generated by the model. No effects of the inhibitory surround were included because we did not test directly the extent of inhibitory surround. In other words, in the model the excitatory RF was represented by a single Gaussian function while in the majority of models two Gaussians represent the excitatory RF center and the inhibitory surround^39^. Our goal was not to explain the mechanisms of adaptation but to identify the adaptation effects on responses to commonly used stimuli, such as moving gratings. We verified that the presence a single Gaussian RF field did not affect the results reported here. First, we verified that the model reproduces well the main observations reported here. Figure 7A shows responses generated by the model when the adaptor and the test stimulus were presented in the same spot at different time intervals between the adaptor and the test stimulus. Apart from few details, simulation results were in good agreement with our experimental data (Fig. 7B–D). The overall time-course of response recovery from locally-induced adaptation in the model matched well the real data, especially when single unit data were considered (Fig. 7B). In the case of non-local adaptation with an adaptor inside RF, where was a slow recovery phase in the experimental data that was absent in the model (Fig. 7C). This discrepancy could be attributed to the partial presence of slowly recovering local adaptation in the experimental data. The spatial extent of local adaptation was adjusted to match typical values of *d*_*50%*_ (compare Figs 4C and 7D and Supplementary Fig. 3). Next we stimulated the model with moving gratings; the results of these simulations are shown in Fig. 7E,F. These results show that adaptation acts as both a temporal and a spatial filter. For instance, in the graph of Fig. 7E there was a clear peak, corresponding to the grating temporal frequency of ~2 Hz. Experimental data from rats show that a number of superior colliculus neurons had very similar temporal tuning^26^. Spatial filtering was limited to higher frequencies: there was strong suppression of responses to gratings with spacing denser than ~0.01 c./deg that can be explained by the spatial extent of local adaptation of about 10 degrees used in the model. Such suppression of higher spatial frequencies is found in all SC neurons in rats^26^. Usually such filtering of higher spatial frequencies is attributed to the limited density of retinal rod or cone cells^40^. However, our model presents an alternative or additional explanation: the extent of local adaptation may be a crucial factor that limits spatial resolution of prolonged visual stimuli. Thus, at least to some extent, adaptation may be responsible for such temporal and spatial filtering of responses to visual stimuli in the SC neurons of rats.

## Discussion

The experiments described here reveal rich spatiotemporal features of adaptation in the superficial layer neurons of the rat SC. A model based on experimental data demonstrated profound filtering effects of adaptation on visual responses of SC neurons.

The main experimental finding is that at least two forms of adaptation affect visual responses in rat SC neurons. The first form of adaptation occurs when an adaptor and a test stimulus are co-localized inside classical RF resulting in diminished both ON and OFF responses, which occur following *bright* stimulus onset and offset correspondingly. We will call this form of adaptation ‘local’ adaptation. It takes several seconds to recover ON responses from local adaptation while OFF responses recover in less than a second. The second form of adaptation occurs when an adaptor stimulus is placed >10° away from the test stimulus, we will call it ‘non-local’ adaptation. The effects of non-local adaptation depend on the adaptor location, inside or outside RF, and the RF size. If an adaptor is placed close to a small RF (<20°), then in the majority of cases the ON responses are enhanced for few hundreds of milliseconds while OFF responses remain unaffected. However, if the adaptor remains inside RF, then ON responses are diminished even when the adaptor is placed >10° away from the test stimulus. In this case ON responses recover quickly, in <0.5 s, while OFF responses remain unaffected. This quick recovery of ON responses and no effect on OFF responses distinguishes the non-local form of adaptation from locally-induced adaptation.

It should be noted, that our method of OFF response elicitation might have introduced a bias in the results: OFF responses were always preceded by a 0.6 s long test stimulus that itself could induce adaptation. However, the adaptation onset graphs in Fig. 3D,E suggest a mono-exponential time-course for both ON and OFF responses indicating that in both cases a single process determined the time-course. Thus, the onset time-course is unlikely to be affected by our choice of stimulation protocols. We also don’t see how this difference in eliciting ON and OFF responses could affect the recovery from adaptation and its spatial spread. Nevertheless, it is plausible that we underestimated the extent of OFF response sensitivity to adaptation since our control OFF responses were already adapted to a 0.6 s long control stimulus. However, the difference between the susceptibility of ON and OFF responses was so large, a ∼3 fold reduction for ON responses and 1/3d depression for OFF responses, that it seems unlikely to be entirely attributable to the stimulation differences. It has been shown that elimination of metabotropic glutamate receptor subtype 6 (mGluR6) in retinal ON bipolar cells leads to the abolishment of ON but not OFF responses in the superior colliculus neurons confirming that in the collicular neurons retinal ON pathway generates ON response while retinal OFF pathway is entirely responsible for OFF responses^41^. In other words, OFF responses in collicular neurons are equivalent to responses induced by light dimming or the onset of dark flashes that are used to specifically activate neurons of retinal OFF pathway^42^. Thus the above mentioned differences in adaptation of ON and OFF responses are likely to originate in the retina.

It is well established that most adaptation to light occurs in the retina^5^. Although adaptation to contrast was entirely attributed to the cortex in early studies^43^; later it has been shown that adaptation to contrast may be entirely derived from the retinal inputs^44^ and is probably related to gain control in the retina^45^. Local adaptation of ON responses described in this paper is likely inherited from the retinal light adaptation because several features of this adaptation matches well the properties of light adaptation in the retinal ganglion neurons^5^. First, fast adaptation onset (<0.1 s, Fig. 5E) and slow recovery of >1 s of local adaptation in SC agrees well with data from the retina^46–48^. Although fast adaptation to contrast also occurs in <0.1 s, we did not see clear evidence for slow adaptation developing in seconds that is so prominent in the retinal contrast adaptation^49,50^. Second, spatial specificity is well within the range of RF sizes of the retinal neurons in rats^30,31,51^. Third, compared to ON responses, adaptors of longer duration were required to induce local adaptation of OFF responses that agrees well with the smaller RF sizes of retinal OFF ganglion cells^52^: since adaptation to light in the retina is proportional to the light flux^53^, to accumulate the same light flux integration times are longer in retinal OFF than ON receptors because RF area of OFF receptors is smaller compared to ON receptors. Meanwhile, recent evidence indicates that OFF bipolar cells generating OFF responses in SCS neurons are more susceptible to contrast adaptation than other bipolar cells^54^. This observation contradicts our data indicating that ON responses are more prone to adaptation than OFF responses. All these considerations suggest that light but not contrast adaptation in the retina is the main source of local adaptation in the rat SC under our experimental conditions.

However, there are at least two features of local adaptation that apparently contradict the hypothesis of the retinal origins. First, the speed of recovery from local adaptation depended on the RF size (Fig. 4G). If we assume that the RF size of a SC neuron is entirely determined in SC, then the speed of recovery from local adaptation inherited from the retina should not correlate with the RF size. Nevertheless, it is plausible that SC neurons possessing small RFs and large RFs receive inputs from distinct populations of retinal ganglion cells that differ in their speed of recovery from adaptation. The second feature of local adaptation that contradicts its retinal origins is the presence of a brief period after the adaptor stimulus when no response to the test stimulus can be detected. In the unit of Fig. 4B, after the gap of 0.05 s, there was neither ON response to the test stimulus, nor the overlapping OFF response to the preceding adaptor stimulus. Under similar stimulation conditions, retinal neurons do respond to changes in light levels even during the onset of adaptation^5^. Nevertheless, it is plausible that a non-linear summation of inhibition and excitation in SC circuitry can explain this phenomenon^55^. Thus, we conclude that local adaptation of ON responses in SC neurons is probably inherited from the retina and a local SC circuitry only modifies the effects of this adaptation.

Non-local adaptation is clearly heterogeneous: the response facilitation or depression depended on both the size of RF and the location of the adaptor stimulus inside or outside RF. When an adaptor stimulus was in RF, ON responses were always suppressed. When an adaptors was outside RF, the adaptation result depended on the RF size. If an adaptor was close to a small RF, responses to stimuli in RF were often facilitated. If an adaptor was close to a large RF, then the result was always no adaptation.

These differences in non-local adaptation of ON responses can be well explained by the normalization model of adaptation that includes the effects of inhibitory surround^4^. According to the model, inside RF the adaptation effects are largely equivalent to the response fatigue with minimal contribution from the normalization pool of the inhibitory surround. Meanwhile, adaptors in the inhibitory surround outside RF will cause adaptation of the inhibitory surround by weakening its suppressive effects on the responses to stimuli inside RF resulting in enhanced responses^4^. Although we did not perform direct verification tests for the presence of an inhibitory surround, we propose that the facilitation of responses was observed when an adaptor activated the inhibitory surround and no facilitation was observed when no activation of inhibitory surround occurred. It is plausible that only the units with small RF possessed an inhibitory surround thus explaining the complete absence of facilitation in the units possessing large RFs.

The main purpose of retinal adaptation is to preserve the full range of neuronal responses under changing light conditions^5^. However, two types of adaptation described here serve more than just a simple mechanism to avoid response saturation. Detailed information on temporal and spatial properties of local and non-local adaptation allowed us to build a simple adaptation model. The results obtained with this model demonstrate that adaptation acts as a powerful spatiotemporal filter, which alone may explain many observed tuning properties of SC neurons^26^. Differences in the adaptation properties between ON and OFF responses suggest their very different functional role, which deserves a separate study. Finally, any visual stimulation paradigm includes multiple presentations of the same or similar visual stimuli. Our results show that often 1 s interval is by far insufficient to recover from local adaptation and the results of such tests may be severely filtered by local form of adaptation.

## Materials and Methods

### Surgical procedures

All procedures were carried out in accordance with the European Communities Council Directive of 22 September 2010 on the protection of animals used for scientific purposes (2010/63/EEC) and were approved by the Animal Care and Use Committee of the State Food and Veterinary Service of Lithuania (No. G2–64 of 31 March 2017).

Standard procedures were used for single unit recordings^22,33^. Briefly, female rats weighting 175 to 250 grams (1.5–2.0 months old) were anesthetised with urethane (Alfa Aesar – Thermo Fisher, Karlsruhe, Germany, 1.2–1.5 g kg^−1^) aided with butorphanol (Richter Pharma AG, Wels, Austria, 0.4 g kg^−1^), both delivered intraperitoneally^56^. The depth of anaesthesia was monitored by testing for the absence of hind limb withdrawal reflex following a pinch of the paw. To maintain the depth of anaesthesia, additional doses of butorphanol were sometimes used for the duration of the experiment. Under anaesthesia, the body temperature was maintained at 36–38 °C with a heating pad. The anesthetized animal was placed in a modified stereotaxic apparatus (World Precision Instruments, Sarasota, FL, USA) that enabled unobstructed view of the left eye. Eye gel was applied to avoid eye drying. Although in anesthetised rats eye movements are rarely a problem^8^, to prevent any eye movements and to maintain lids open, miniature hooks were inserted between the conjunctiva of the inner eyelids and the sclera of the eyes and then attached to the stereotaxic frame with a thread. To dilate eyes, atropine (Sigma-Aldrich Chemie GmbH, Taufkirchen, Germany) solution of 0.5% was applied to the eye surface. Eye dilation increases light scattering which may confound the measurements of RF size and adaptation spatial spread. However, pupil dilation or systemic atropine, which also results in eye dilation, is often applied by researchers in the field^24,25^, making the comparison of our data with other studies straightforward. Moreover, the estimated adaptation spatial spread agrees well with the retinal RF size measurements, indicating that light scattering was not a major problem under our experimental conditions.

For recording, a small craniotomy (approximately 2 × 2 mm) was made in the parietal bone. Dura mater was left intact. Tetrodes from Thomas Recording (Giessen, Germany) were used to acquire data. Electrodes were typically placed 1.8–2.2 mm rostral to liambda and 1.4–2.2 mm lateral to the midline and then lowered perpendicular to the cortical surface by means of a micro-drive to a depth of >~2000 µm. The presence of the superior colliculus was identified by characteristic spontaneous activity and robust responses to visual stimuli such as movements of small bright spots. For data acquisition a 4-channel differential amplifier was used (EX4–400, DAGAN, Minneapolis, MN, USA) with a band-pass filters set to 300–10,000 Hz. Data were acquired via a National Instrument card to a PC at 40 kHz sampling frequency and visualized by employing a custom program written in the Labview environment (National Instruments, Austin, TX, USA).

### Visual stimulation

A LED backlit LCD monitor (frame rate 60 Hz, 58 cm by 28 cm) was used for image presentation and was placed 16 cm from the rat right eye slightly below rat’s plane at 45 degrees angle to the rat’s longitudinal axis in the horizontal plane. In the vertical plane the computer monitor was inclined at 30 degrees towards the rat in order to cover a wider range of vertical angles. The full screen subtended 110 horizontally and about 80 degrees vertically. At the center of the screen 1 cm corresponded to ~3.6° of visual angle. During experiments the stimulus size was entered as a fraction of the monitor height; 1/10^th^ of the monitor height being considered to correspond to 10° of visual angle (actual calculations show that it is 2.8 cm * 3.4 deg/cm = 10.08 deg). Rat eyes are normally emmetropic and can see sufficiently well from 7 cm to infinity^57^. The monitor had 1920 × 1080 image pixels or >30 pixels/cm corresponding to a minimal stimulus size of <0.1°, well below visual acuity of ~0.5°–1° found in rodents^58^. Images were generated by employing an open source software package PsychoPy controlled by an in house program written in the Labview environment (National Instruments, Austin, TX, USA)^59^.

All visual stimuli were bright images, ~30–45 lx at rat’s eye level, presented on a dark grey background (~0.35–0.45 lx).

The following visual stimulation protocols were used.

1. The size and location of RF was determined by flashing 2.5°–3.0° wide bright round spots for 600 ms followed by a 900 ms gap (1.5 s inter-stimulus interval, ISI) on a 18 × 11 grid in a quasi-random fashion. The grid spacing corresponded to 7.5° and covered most of the monitor. The size of RF was estimated by fitting with a two-dimensional Gaussian distribution separately for ON and OFF spike rate values on the grid^25^:
  1 $$G(x,y)=\frac{A}{2\pi ab}\exp (\frac{{X}^{\text{'}2}}{2{a}^{2}}+\,\frac{{y}^{\text{'}2}}{2{b}^{2}}),$$
  where *x*′ and *y*′ are the polar transformations of space coordinates x and y at an angle θ, along which the Gaussian distribution is oriented. The RF diameter was estimated as a square root of a sum of squared parameters *a* and *b* obtained during the fit procedure.
2. To determine adaptation to multiple stimulus presentations, bright spots 2.5° in diameter were flashes for 600 ms followed by a 900 ms pause (1.5 s ISI). The selected test stimulus duration of 600 ms was minimal to achieve reliable separation of ON and OFF responses in the majority of recorded units.
3. To determine the time-course of recovery from adaptation, the conditioning adaptor stimulus was presented each 30 s and then it was followed by a test stimulus after a 0.05 s, 0.1 s, 0.2 s, 0.4 s, 0.8 s, 1.6 s, 3.2 s, 6.4 s and 12.8 s gap. At the start of each cycle, which included all listed gaps in increasing order, two control tests were performed: a conditioning stimulus was presented alone and then 31 s later a test stimulus was presented. Responses to these two tests were used as control responses to the adaptor and test stimulus correspondingly. Usually an alternation of adaptor co-localized with the test stimulus and shifted from the test stimulus was used. In this case, first a full cycle with two controls and nine different pauses was run for the adaptor shifted from the test stimulus location and then a full cycle of adaptor co-localized with the test stimulus was run. At least 8 such alternations were used to obtain the recovery data.
4. The spatial specificity of adaptor effects was determined by presenting 4 times the adaptor stimulus and then twice the test stimulus. The use four presentations of adaptor stimulus instead of one resulted in a stronger adaptation effects and permitted to increase the sensitivity of the paradigm. These tests included a control series, in which the adaptor and the test stimulus locations were the same. This series was used to determine the fraction of response sensitive to adaptation that was defined as a difference in AP frequency between the response to the first adaptor stimulus and the 5^th^ stimulus. In other series, when the adaptor location was shifted from the test location, the 5^th^ stimulus was the first test stimulus. Then the adaptor location was gradually shifted from the test stimulus location until the response to the first test stimulus reached the same or almost the same amplitude as to the first stimulus in the control series. Then the spike rate count for each location was plotted against the distance from the test stimulus location. These results were fit with a sigmoid function:
  2 $$S(d)=\frac{A}{(1+\exp \frac{({d}_{50 \% }-d)}{rate})},$$
  where *S* is spike rate during ON or OFF response, d is the distance from the test stimulus location, *A* is the maximal spike rate, *d*_*50%*_ is the distance from the test stimulus site, at which half of the original spike rate is reached, *d*_*50%*_ is used throughout this paper as an estimate of adaptation spatial specificity, *rate* is the slope of spike rate increase with distance, in this paper it was used only as a fit parameter.
5. The effects of adaptor duration were determined by presenting adaptor stimulus of varying duration followed by the test stimulus of 0.6 s. Adaptors were presented each 30 s followed by 3 different gap durations between the adaptor and the test stimuli. Three different gap durations were employed: 0.1 s, 0.8 s and 6.4 s. Five different durations of the adaptor stimulus were used, 0.05 s (3 monitor frames), 0.1 s, 0.25 s, 0.6 s and 1.5 s.
6. To determine optimal stimulus size, stimuli of different sizes from 0.1° to 20° were presented each 20 s.

### Data Analysis

Most analysis on recorded traces was performed with custom written routines by employing Igor Pro software (Wavemetrics, Lake Oswego, Oregon, USA). Matlab (Mathworks, Natick, MA, USA) was used for principal component analysis (PCA) while data clustering analysis was performed with a publically available KlustaKwik software (http://klustakwik.sourceforge.net).

*Spike detection and sorting*. Single units were first detected as threshold-crossing events while the threshold was set from 3 to 4.5 standard deviations from the baseline. The standard deviation was calculated according to Quiroga *et al*.^60^. The threshold was lowered by 30% when synchronous events occurred on 2 or more traces. Care was taken that no artifacts were present in the trace. For each detected event between 1.1 ms and 1.6 ms of each channel data were collected, 44–62 data points in total, 14–20 before and 30–45 after the negative peak. The window duration was determined by the shape of action potentials. For spike sorting, first each channel data were reduced by principal component analysis (PCA) by employing Matlab software. Then spike classes were determined by finding clusters of 2–5 PCA from each channel by employing KlustaKwik software (http://klustakwik.sourceforge.net). If the results were unsatisfactory according to the absence of sufficient refractory period or poor cluster separation^61^, additional feature based clustering was performed. The quality of sorting was verified with auto- and cross-correlograms^61^.

All results are presented as average ± SEM. Unless stated otherwise, a non-parametric Kruskal-Wallis test was used for statistical significance.

### Data Availability

The authors declare that all data necessary for evaluation of our statements is present in the manuscript and supplementary information files. However, any additional reasonable requests for data will be granted by the corresponding author.

## Electronic supplementary material

Supplementary information
