# Supplementary material for: Experimentally derived model shows that adaptation acts as a powerful spatiotemporal filter of visual responses in the rat collicular neurons
Source: Sci Rep. 2018 Jun 12;8:8942. doi: 10.1038/s41598-018-27331-2 (PMC5997664; doi:10.1038/s41598-018-27331-2)
Supplement: Supplementary file 1 — Supplementary information [file 41598_2018_27331_MOESM1_ESM.pdf]

Supplementary information

Title:

Experimentally derived model shows that adaptation acts as a powerful spatiotemporal filter of visual responses in the rat collicular neurons

Juntaute Bytautiene[1], Gytis Baranauskas[2*]

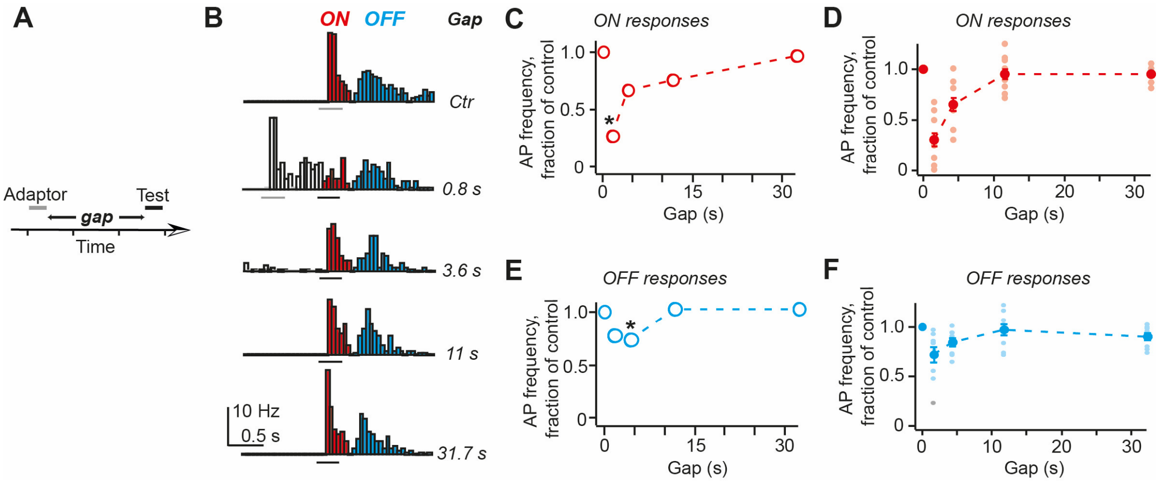

Supplementary Figure 1. Most response amplitude was recovered from adaptation within 11 s. *A*. Schematics of the stimulation protocol. The protocol was repeated each 90 s. *B*. PSTHs of a representative unit are shown for responses to the test stimulus (a black bar below PSTH) following 0.8 s, 3.6 s, 11 s and 31.7 s gap after the adaptor stimulus (a grey bar below PSTH). PSTH bars of ON responses are red while OFF response bars are shown in blue. Unrelated activity is shown as open bars. *B*. The time course of recovery of ON responses of the unit shown in *A*. *C*. The summary of all tested unit ON responses. Averages + SEM are shown in dark red while individual unit data are shown in light red. *D*. The time course of recovery of OFF responses of the unit shown in *A*. *E*. The same as *C* but for OFF responses.

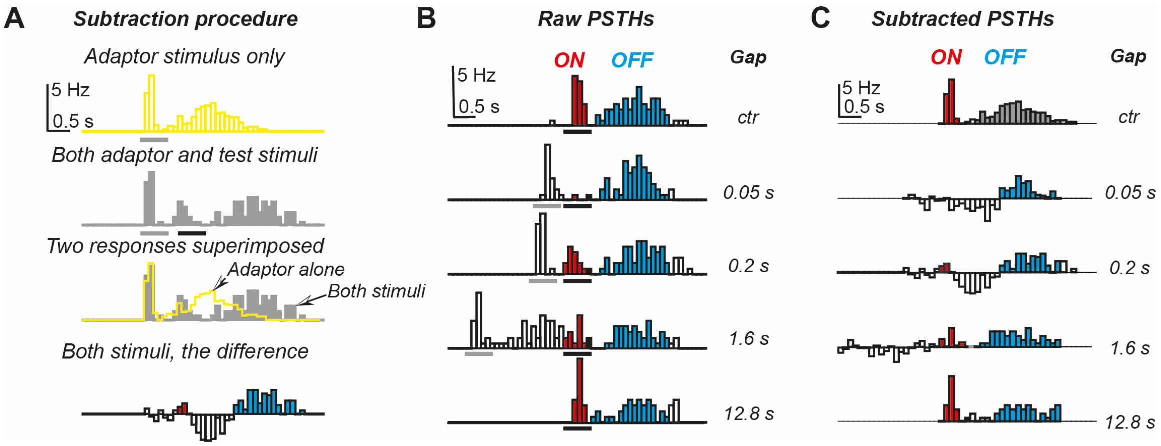

Supplementary Figure 2. The outline of the general procedure used to subtract the response to adaptor from the response to the stimulus. *A*. First, an adaptor stimulus was presented alone (yellow bars in the first row) and then in combination with a test stimulus (grey bars in the second row). The estimated contribution of the adaptor stimulus was subtracted from the total response to the adaptor followed by the test stimulus (both responses are over-lapped in the 3d row) and the remaining response after subtraction was considered to be the response to the test stimulus (the bottom row with red and blue bars indicating ON and OFF responses correspondingly). If the result of subtraction was negative (open bars below the 0 line), it was considered that the tests stimulus produced no response. To obtain ON or OFF responses, these negative data bins obtained after subtraction were forced to 0 and only then the integral of ON and OFF responses was calculated. *B*. Original, non-subtracted PSTHs of a unit shown in figure 2. *C*. Subtracted PSTHs of the same unit.

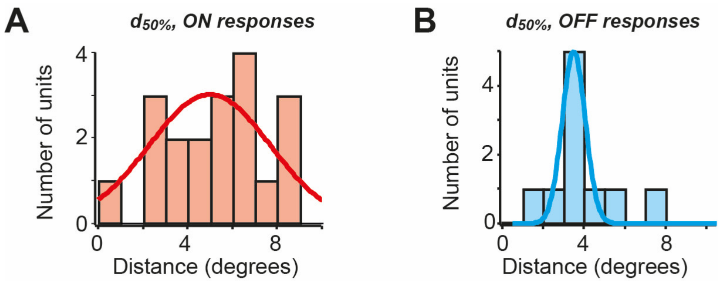

Supplementary Figure 3. The distribution of d50% for ON (*A*) and OFF (*B*) responses. Continuous lines represent Gaussian function fits.

Adaptation model

First, we should note that our model mostly represents a simplified version of many traditional retinal light adaptation models. Typically, in a model, light adaptation is introduced as a low-pass filtered feedback for gain control, often two stages of feedback are used [1-4]. Meanwhile, in the traditional models RF is represented as a difference-of-Gaussians and the output is often obtained as a low-pass filtered impulse response [5,6]. Our model includes all these elements except for a single Gaussian function that was used to represent the excitatory RF center while no second Gaussian for the inhibitory surround was added. The two stages of the feedback for the gain control are lumped into a single equation as explained below. In addition, in contrast to most retinal light adaptation models, our simulations included two types of adaptation mechanisms, locally and non-locally-induced. These two mechanisms were represented by two different parameters in the same gain-control equation. Computational details of the model are provided below.

Neuronal responses were generated in two stages, response function formation with linear transforms and then filtration and rectification of the response function. The response function was obtained by modifying the canonical normalization equation, which can be written as [7]:

$$R = \gamma \frac{I^2}{\sigma^2 + I^2}, \qquad (1)$$

where R is the response function, γ is a constant determining the maximal response, *I* is the light flux, which is calculated according to the procedure described below, σ is a parameter, which determines saturation and adaptation effects of responses, the exact definition is provided below.

Equation (1) resembles the Naka – Rushton relation [8] with one major difference: in equation (1) light flux *I* is squared while in the Naka – Rushton equation it is not. This difference is to a certain degree equivalent to having two stages of feedback control: in steady state conditions a single stage would yield Naka – Rushton equation while two stages correspond to a squared yield, which can be transformed to equation (1). Since we wanted to include two types of adaptation, for which light fluxes were calculated in a different manner, equation (1) was slightly modified to have both the dividend and the divisor dimensionless:

$$R = \gamma \frac{1}{\frac{\sigma^2}{I^2} + 1} \qquad (2)$$

Our experimental data showed, that for adaptors located within RF two forms of adaptation should be induced:

1) Local adaptation; the recovery from this form of adaptation was slow and often no response could be detected for brief intervals between the adaptor and the test stimulus;

2) Non-local adaptation when an adaptor placed within RF was able to reduce responses to visual stimuli located in any area within RF, the recovery from this form of adaptation was much faster and the responses were never completely abolished.

Since the latter form of adaptation did not abolish responses, in the model it was introduced as a modifier of the quotient σ^2/I^2 in equation (2):

$$R = \gamma \frac{1}{\left(1+3*\frac{\sigma_{RF}^2}{I_{RF}^2}\right)*\frac{\sigma_{loc}^2}{I_{loc}^2}+1}, \quad (3)$$

where I_{RF} is the light flux within RF, calculated as a single Gaussian, while I_{loc} is the local light flux, which drives local adaptation; equations for both light fluxes are provided below. Parameters σ_{RF} and σ_{loc} determine non-local and local adaptation correspondingly, equations defining them are given below. A factor of 3 in the divisor of equation (3) was introduced to reflect the fact that in non-locally induced adaptation (marked as RF in equation (3)) the response amplitude was reduced to 1/4th of the control. To calculate I_{RF} and I_{loc}, the following equation was used:

$$I_{total} = \sum_{xy} a_{xy} * I_{xy}, \quad (4)$$

where I_{total} is the total RF or local light flux, I_{xy} is the light flux at (*x, y*) coordinates of the visual field plane and a_{xy} is an element of a normalized matrix of RF/local light field. A 50 by 50 grid with 1 degree spacing was used to calculate light fluxes. A two-dimensional Gaussian function (equation (1) in the Methods section) was applied to calculate both the RF and the local light matrix coefficients a_{xy}. It was assumed that the RF diameter was 10 degrees in both *x* and *y* directions while for local light flux the diameter of the local light flux was 2.5 degrees in both directions. The parameters σ_{RF} and σ_{loc} were calculated by employing a simple differential equation:

$$\sigma_{i+1} = \sigma_i + (I_i - \sigma_i) * \frac{dt}{\tau_{onset}} - \sigma_i * \frac{dt}{\tau_{offset}}, \quad (5)$$

where dt is a 0.01 s step, τ_{onset} is the time constant of adaptation onset, equal to 0.1 s in both cases; τ_{offset} is the time constant of recovery from adaptation, set to 0.4 s for non-local and 1 s for local adaptation. Responses of the model were obtained by first filtering the response function of equation (3) by employing equation (5), in which τ_{onset} was set to 0.03 s and τ_{offset} to 0.2 s (this stage corresponds to photoreceptor response function formation) and then rectifying the obtained result at a threshold of 0.3. We verified that this large stage of filtering did not contribute to the results shown in figure 7.

1 Shah, S. & Levine, M. D. Visual information processing in primate cone pathways. I. A model. *IEEE Trans Syst Man Cybern B Cybern* **26**, 259-274, doi:10.1109/3477.485837 (1996).

2 Crevier, D. W. & Meister, M. Synchronous period-doubling in flicker vision of salamander and man. *J Neurophysiol* **79**, 1869-1878, doi:10.1152/jn.1998.79.4.1869 (1998).

3 Hood, D. C. Lower-level visual processing and models of light adaptation. *Annu Rev Psychol* **49**, 503-535, doi:10.1146/annurev.psych.49.1.503 (1998).

4 Snippe, H. P., Poot, L. & van Hateren, J. H. A temporal model for early vision that explains detection thresholds for light pulses on flickering backgrounds. *Vis Neurosci* **17**, 449-462 (2000).

5 Donner, K. & Hemila, S. Modelling the spatio-temporal modulation response of ganglion cells with difference-of-Gaussians receptive fields: relation to photoreceptor response kinetics. *Vis Neurosci* **13**, 173-186 (1996).

6 Einevoll, G. T. & Plesser, H. E. Response of the difference-of-Gaussians model to circular drifting-grating patches. *Vis Neurosci* **22**, 437-446, doi:10.1017/s0952523805224057 (2005).

7 Carandini, M. & Heeger, D. J. Normalization as a canonical neural computation. *Nat Rev Neurosci* **13**, 51-62, doi:10.1038/nrn3136 (2011).

8 Shapley, R. M. & Enroth-Cugell, C. Visual adaptation and retinal gain control. *Prog Ret Res* **3**, 263-346 (1984).
